# Comparative Evaluation of GeneXpert With Ziehl-Neelsen (ZN) Stain in Samples of Suspected Tuberculosis Cases at a Tertiary Care Teaching Hospital in Central India

**DOI:** 10.7759/cureus.71402

**Published:** 2024-10-13

**Authors:** Jyoti Gupta, Priyanka Joshi, Rajesh Gupta, Vikas Gupta

**Affiliations:** 1 Microbiology, Mahaveer Institute of Medical Sciences and Research, Bhopal, IND; 2 Pulmonary Medicine, Mahaveer Institute of Medical Sciences and Research, Bhopal, IND; 3 Pediatrics, Datia Medical College, Datia, IND; 4 Community Medicine, Government Medical College, Seoni, IND

**Keywords:** diagnostic efficacy, extrapulmonary tb, genexpert mtb/rif, pulmonary tb, ziehl-neelsen staining

## Abstract

Background

Tuberculosis (TB) remains a significant public health challenge, particularly in developing countries, where delayed diagnosis contributes to ongoing transmission. Ziehl-Neelsen (ZN) smear microscopy, commonly used for TB diagnosis, has limitations in sensitivity, especially in cases of extrapulmonary tuberculosis (ETB). The GeneXpert *Mycobacterium tuberculosis* (MTB)/rifampicin (RIF) assay, a molecular diagnostic tool, offers rapid and accurate detection of MTB and RIF resistance. This study aimed to compare the diagnostic efficacy of GeneXpert MTB/RIF with ZN staining in detecting pulmonary tuberculosis (PTB) and ETB.

Methods

A prospective study was conducted over two years (April 2022 to April 2024) at a tertiary care teaching hospital in India. A total of 319 clinical samples from patients with suspected PTB and ETB were analyzed. Samples underwent ZN staining and GeneXpert MTB/RIF assay. Sensitivity, specificity, positive predictive value (PPV), and negative predictive value (NPV) were calculated using mycobacterial culture as the gold standard. The chi-square test was employed to compare diagnostic accuracy, with a p-value of <0.05 considered statistically significant.

Results

Of the 319 samples, ZN staining was positive in 18.2% of cases, while GeneXpert was positive in 21.6%. GeneXpert demonstrated a perfect sensitivity of 100% and a specificity of 98.81%, compared to ZN staining’s sensitivity of 84.85% and specificity of 99.21%. GeneXpert showed superior performance in detecting TB in both pulmonary and extrapulmonary samples, with a statistically significant difference (p<0.001). Additionally, GeneXpert identified six cases of RIF resistance.

Conclusion

The GeneXpert MTB/RIF assay outperforms ZN staining in diagnosing TB, offering higher sensitivity and comparable specificity. Its ability to detect RIF resistance adds significant clinical value. Despite its cost, the integration of GeneXpert into routine diagnostic workflows, particularly in high TB prevalence areas, is recommended to enhance early detection and treatment, thereby reducing TB transmission.

## Introduction

Tuberculosis (TB) is one of the oldest diseases known to affect humans and remains a significant public health challenge globally [1]. Caused by Mycobacterium tuberculosis (MTB), TB is the leading cause of death from a single infectious agent. According to the Global TB Report 2023, an estimated 10.6 million incidents of TB were reported in 2022 [2,3]. The disease poses a considerable risk of transmission, as a single active case can infect several individuals before receiving treatment, creating a continuous reservoir of TB-infected individuals [4].

Early and accurate diagnosis is crucial for effective management and improving patient outcomes. However, TB diagnosis often faces significant challenges, especially in developing nations. Traditional methods, such as Ziehl-Neelsen (ZN) smear microscopy, have poor sensitivity and can lead to false-negative results. This technique requires multiple visits and has been associated with higher default rates due to its limitations [5,6]. While mycobacterial culture is considered the gold standard, it is slow, typically taking two to six weeks to produce results, and requires substantial infrastructure and technical expertise [7].

In December 2010, the World Health Organization (WHO) endorsed the GeneXpert MTB/RIF assay for diagnosing TB and multidrug-resistant tuberculosis (MDR-TB) [8]. The GeneXpert assay employs a DNA-PCR technique for the simultaneous detection of MTB and mutations related to rifampicin (RIF) resistance. It is the first fully automated benchtop cartridge-based nucleic acid amplification test (CB-NAAT) for TB detection, providing results within two hours and offering high diagnostic accuracy for pulmonary tuberculosis (PTB) [9,10].

Diagnosing extrapulmonary tuberculosis (ETB) presents additional challenges due to the low number of MTB bacilli in affected tissues and difficulties in obtaining adequate clinical specimens from deep-seated organs. The complexity of investigating EPTB includes diverse clinical sample types, challenges in obtaining sufficient tissue for analysis, and issues with extracting MTB DNA from samples [5,8,10].

Given these challenges, this study aims to compare the diagnostic efficacy of the GeneXpert MTB/RIF assay with ZN staining (smear and microscopy) for diagnosing PTB at a tertiary care teaching hospital in Central India. This comparison seeks to provide valuable insights into the performance of GeneXpert in a specific setting and its potential to improve TB diagnosis and management.

## Materials and methods

Study design and setting

This prospective cross-sectional study was conducted in the Department of Microbiology at Mahaveer Institute of Medical Sciences and Research (MIMS), Bhopal, India, spanning approximately two years from April 2022 to April 2024.

Study participants and sample size

Patients clinically suspected of having PTB or ETB based on symptoms and preliminary findings were included. PTB suspicion was based on symptoms such as persistent cough lasting over two weeks, fever, night sweats, weight loss, and hemoptysis. ETB suspicion included symptoms like lymphadenopathy, pleural effusion, or abdominal pain. Participants ranged from pediatric to elderly patients. Clinical suspicion was supported by preliminary tests, including chest X-rays or ultrasounds and initial laboratory tests. Exclusion criteria included lack of clinical history, patients with lung malignancies or fungal infections, and those who had received anti-TB treatment in the past two months.

Sample collection

A total of 319 samples were collected from suspected TB patients. For PTB, sputum or bronchoalveolar lavage (BAL) samples were obtained. Extrapulmonary samples included pleural fluid, pus, and tissue. All samples were transported under cold chain conditions in triple-layer packing to designated laboratories for processing.

Laboratory methods

Samples were processed using ZN staining and the GeneXpert MTB/RIF assay. For sputum samples, direct smears were prepared, air-dried, heat-fixed, and stained using the ZN method, with grading according to National Tuberculosis Elimination Program (NTEP) guidelines [11]. Tissue samples were homogenized or torn apart, smears prepared, and stained similarly with the ZN method. In the GeneXpert MTB/RIF assay, sputum samples were mixed with a sample reagent (2:1 ratio), agitated, and incubated at room temperature for 10 minutes. Two milliliters of the processed sample were added to a GeneXpert cartridge for analysis, with results available after approximately 90 minutes. Tissue samples were chopped, mixed with a 2:1 volume of reagent buffer, and processed similarly. The GeneXpert assay was conducted in a BSL-3 laboratory, adhering to standard precautions and Cepheid guidelines, with only electronic results used for comparison [12].

Statistical analysis

Statistical analysis was performed using SPSS version 26.0 (IBM Corp., Armonk, NY, USA). Descriptive statistics summarized patient demographics and clinical characteristics. Sensitivity, specificity, positive predictive value (PPV), and negative predictive value (NPV) of ZN smear microscopy and GeneXpert MTB/RIF assay were calculated, using mycobacterial culture as the gold standard. Diagnostic accuracy was compared using the chi-square test, with a p-value of <0.05 considered statistically significant.

Ethical considerations

The study was approved by the Institutional Ethics Committee (Approval number: MIMSR/IEC/2022/3/008). Written informed consent was obtained from all participants or their guardians before sample collection. The confidentiality of patient data was maintained throughout the study.

## Results

A total of 319 clinical samples were analyzed, with a mean participant age of 38.6±20.5 years, and a predominant male population (76.4%). Most samples were pulmonary (95.3%), primarily sputum (93.2%), while extrapulmonary samples comprised 4.7%, with pus being the most common (2.8%). ZN staining was positive in 18.2% of cases, GeneXpert in 21.6%, and culture (the gold standard) in 20.7%. The GeneXpert assay also identified six cases of RIF resistance (Table 1).

**Table 1:** Baseline Characteristics of Study Participants (N=319). BAL, bronchoalveolar lavage; ZN, Ziehl-Neelsen

Characteristic	Frequency (%)/mean±SD	%
Age (years)	38.6±20.5	
Age group (years)		
<20	65	20.4
21-30	68	21.3
31-40	32	10.1
41-50	36	11.3
51-60	69	21.6
>60	49	15.3
Gender		
Male	244	76.4
Female	75	23.6
Type of sample		
Pulmonary	304	95.3
Sputum	297	93.2
BAL	7	2.2
Extrapulmonary	15	4.7
Pus	9	2.8
Tissue	5	1.6
Pleural fluid	1	0.3
ZN staining		
Positive	58	18.2
Negative	261	81.8
GeneXpert		
Positive	69	21.6
Negative	250	78.4
Culture		
Positive	66	20.7
Negative	253	79.3

ZN staining detected MTB in 17.4% of pulmonary and 33.3% of extrapulmonary samples, though this difference was not statistically significant (p=0.119). In contrast, GeneXpert detected MTB in 19.7% of pulmonary and 60.0% of extrapulmonary samples, with a significant p-value of 0.0002, indicating higher sensitivity for extrapulmonary cases (Table 2).

**Table 2:** Comparison of GeneXpert and ZN Staining for Pulmonary and Extrapulmonary Samples (N=319). ZN, Ziehl-Neelsen

Sample type	Frequency (%)		P-value
	ZN stain positive (n=58)	ZN stain negative (n=261)	
Pulmonary (n=304)	53 (17.4)	251 (82.6)	0.119
Extrapulmonary (n=15)	5 (33.3)	10 (66.7)	
	GeneXpert positive (n=69)	GeneXpert negative (n=250)	
Pulmonary (n=304)	60 (19.7)	244 (80.3)	0.0002
Extrapulmonary (n=15)	9 (60.0)	6 (40.0)	

Diagnostic performance of ZN stain

In this study, a total of 319 clinical samples were analyzed using the ZN stain and GeneXpert assay. Of the 66 culture-positive samples, ZN staining was positive in 56 cases (84.85% sensitivity) and negative in 10 cases (Table 3).

**Table 3:** Diagnostic Performance of ZN Staining Compared to Culture Results (N=319). ZN, Ziehl-Neelsen

Diagnostic test	Culture positive (n=66)	Culture negative (n=253)	Total (n=319)
ZN stain			
Positive (n=58)	56	2	58
Negative (n=261)	10	251	261
Total	66	253	319

Among the 253 culture-negative samples, ZN staining was positive in two cases and negative in 251 cases, resulting in a specificity of 99.21%. The ZN stain demonstrated high specificity but relatively lower sensitivity compared to GeneXpert. The positive likelihood ratio was 107.33, while the negative likelihood ratio was 0.15. The PPV of ZN staining was 96.55%, and the NPV was 96.17%, with an overall accuracy of 96.24% (Table 4).

**Table 4:** Diagnostic Metrics of ZN Staining Compared to Culture Results (N=319). PPV, positive predictive value; NPV, negative predictive value; ZN, Ziehl-Neelsen

Metric	Value	95% CI
Sensitivity	84.85%	73.90% to 92.49%
Specificity	99.21%	97.17% to 99.90%
Positive likelihood ratio	107.33	26.89 to 428.44
Negative likelihood ratio	0.15	0.09 to 0.27
PPV	96.55%	87.52% to 99.11%
NPV	96.17%	93.41% to 97.80%
Accuracy	96.24%	93.52% to 98.04%

Diagnostic performance of GeneXpert

GeneXpert testing revealed a high diagnostic capability in this study. Out of 66 culture-positive samples, GeneXpert was positive in 66 cases (100% sensitivity), while no culture-negative samples tested positive (Table 5).

**Table 5:** Diagnostic Performance of GeneXpert MTB/RIF Assay Compared to Culture Results (N=319). MTB, *Mycobacterium tuberculosis*; RIF, rifampicin

Diagnostic test	Culture positive (n=66)	Culture negative (n=253)	Total (n=319)
GeneXpert			
Positive (n=69)	66	3	69
Negative (n=250)	0	250	250
Total	66	253	319

Among the 253 culture-negative samples, GeneXpert was negative in 250 cases, resulting in a specificity of 98.81%. The positive likelihood ratio for GeneXpert was 84.33, with a negative likelihood ratio of 0.00, indicating its superior performance in detecting MTB. The PPV was 95.65%, and the NPV was 100%, achieving an overall accuracy of 99.06% (Table 6).

**Table 6:** Diagnostic Metrics of GeneXpert MTB/RIF Assay Compared to Culture Results (N=319). PPV, positive predictive value; NPV, negative predictive value; MTB, *Mycobacterium tuberculosis*; RIF, rifampicin

Metric	Value	95% CI
Sensitivity	100.00%	94.56% to 100.00%
Specificity	98.81%	96.57% to 99.75%
Positive likelihood ratio	84.33	27.38 to 259.73
Negative likelihood ratio	0	-
PPV	95.65%	87.72% to 98.55%
NPV	100.00%	98.54% to 100.00%
Accuracy	99.06%	97.28% to 99.81%

Comparison of ZN stain and GeneXpert results

When comparing the results of ZN staining and GeneXpert assay, ZN staining detected MTB in 52 of the 69 samples positive by GeneXpert, while six GeneXpert-positive samples were ZN staining-negative. For the GeneXpert-negative group, 17 samples were ZN staining-positive, and 244 were ZN staining-negative. This comparison highlights that while GeneXpert is more sensitive and specific, ZN staining still contributes to the overall diagnostic process, though it missed some cases detected by GeneXpert (Table 7).

**Table 7:** Comparison of ZN Staining and GeneXpert MTB/RIF Assay Results (N=319). MTB, *Mycobacterium tuberculosis*; RIF, rifampicin; ZN, Ziehl-Neelsen

ZN stain result	GeneXpert positive (n=69)	GeneXpert negative (n=250)	Total (n=319)
Positive (n=58)	52	6	58
Negative (n=261)	17	244	261
Total	69	250	319

## Discussion

India bears a significant burden of TB, accounting for approximately one-fourth of global cases [13]. Traditional methods, such as acid-fast bacilli (AFB) detection through ZN staining, although simple, rapid, and specific for diagnosing PTB, suffer from low sensitivity [14]. In contrast, molecular diagnostic techniques, such as the Cepheid GeneXpert system, have revolutionized TB detection by providing rapid, highly sensitive, and specific results. The availability of GeneXpert testing at district and taluka levels under the NTEP has greatly enhanced TB diagnosis across India, making it accessible to a broader population and free of cost to all patients [15].

In our study, among 319 samples from suspected PTB and ETB cases, ZN staining was positive in 18.2% of cases, whereas GeneXpert was positive in 21.6%, demonstrating superior diagnostic capability. Notably, GeneXpert also identified six cases of RIF resistance (Table 1). These findings are consistent with Chinedum et al., who reported GeneXpert positivity in 65.7% of cases compared to 38.6% for smear examination [15]. Similarly, Bajrami et al. found that GeneXpert detected MTB in 29.3% of cases, whereas ZN staining was positive in only 14.6% [16].

The sensitivity of ZN staining in our study was 84.85%, and its specificity was 99.21%, aligning with previous reports such as those by Dzodanu et al. and Chen et al., which highlight ZN staining's high specificity but lower sensitivity compared to molecular methods [17,18]. The low sensitivity of ZN staining is largely due to its dependence on visual detection of AFB, which can be influenced by sample quality and bacterial load [19].

In contrast, GeneXpert showed perfect sensitivity (100%) and high specificity (98.81%) in our study, with an overall accuracy of 99.06%. These findings are consistent with studies by Banerjee et al., Yadav et al., and Osei Sekyere et al., which emphasize GeneXpert's superior sensitivity, especially for detecting MTB in extrapulmonary samples [20-22]. For example, Chaudhary et al. reported a GeneXpert sensitivity of 96.0%, significantly higher than the 46.0% sensitivity of smear microscopy, with GeneXpert also detecting RIF resistance [14]. Kohli et al. similarly noted GeneXpert's high sensitivity (94%) and specificity (98%) for PTB, with even greater sensitivity in extrapulmonary cases [23]. The perfect sensitivity observed in our study underscores GeneXpert's ability to detect even low quantities of mycobacterial DNA, which is critical for early diagnosis and treatment.

For pulmonary samples, our study found ZN staining positivity in 17.4% of cases, while GeneXpert detected MTB in 19.7%, similar to findings by Agarwal et al. [24]. In comparison, Yadav et al. reported a 47.7% positivity rate using GeneXpert and a 23.6% rate with ZN staining for pulmonary samples [25].

For extrapulmonary samples, GeneXpert demonstrated a 60.0% positivity rate compared to 33.3% for ZN staining. This aligns with the growing body of evidence suggesting that GeneXpert is more effective for diagnosing ETB, where ZN staining's efficacy is often limited by lower bacterial loads [26]. The molecular approach of GeneXpert, which targets pathogen DNA, offers a clear advantage over traditional staining methods, as recommended by the WHO in 2012 for routine use under programmatic conditions [27]. Yadav et al. found GeneXpert positive in 49.1% of extrapulmonary cases compared to 11.9% positivity with ZN microscopy [25].

Interestingly, we observed that six sputum samples positive for AFB on ZN staining tested negative for MTB on GeneXpert. These cases likely represent infections with nontuberculous mycobacteria (NTM), as GeneXpert specifically detects the DNA of the MTB complex and not NTM [14,24].

In the context of India, where TB remains a major public health challenge, the widespread implementation of GeneXpert faces several obstacles, especially in rural and resource-limited settings. Although the NTEP has made significant strides by installing GeneXpert machines in many district hospitals, many rural health centers still lack access due to infrastructural limitations [24]. Frequent power outages, lack of temperature-controlled environments, and inadequate technical expertise are common in these areas, hindering the effective use of GeneXpert. Additionally, the cost of each test cartridge (ranging from ₹700-900) and the high upfront cost of the machine make routine testing financially unviable for many smaller clinics and health centers operating on tight budgets [25].

To address these challenges, it is crucial to adopt innovative solutions, such as mobile diagnostic vans equipped with GeneXpert machines, which can reach remote areas lacking healthcare infrastructure. Furthermore, public-private partnerships can play a key role in subsidizing costs, ensuring that even the most marginalized communities have access to advanced TB diagnostics. Expanding training programs for local healthcare workers to operate and maintain GeneXpert systems, coupled with government initiatives to improve infrastructure, can enhance its feasibility in rural India. Such efforts would be instrumental in bridging the diagnostic gap, ultimately accelerating India's progress toward the goal of TB elimination by 2025 [23].

Limitations and future directions

While our study underscores the effectiveness of GeneXpert in diagnosing TB, several limitations warrant consideration. First, we did not differentiate between MTB and NTM in cases where ZN staining was positive but GeneXpert was negative, which limits our ability to interpret the specificity of ZN staining in these instances. Additionally, the potential influence of variations in bacterial load on the sensitivity of GeneXpert was not fully assessed, possibly leading to an underestimation of its diagnostic accuracy, particularly in low-bacillary samples. The absence of confirmatory molecular techniques, such as PCR or line probe assay, for cases with discrepant results further restricts our ability to draw definitive conclusions about the comparative diagnostic performance. Furthermore, we did not comprehensively evaluate the feasibility and cost-effectiveness of using both ZN staining and GeneXpert in resource-limited settings, which could provide valuable insights for practical implementation. Finally, the small number of discrepant cases in our study limits the statistical power to fully understand the observed differences in diagnostic outcomes.

## Conclusions

This prospective study evaluated the diagnostic efficacy of the GeneXpert MTB/RIF assay compared to ZN staining for TB detection in a tertiary care hospital in Central India. The GeneXpert assay demonstrated superior sensitivity (100%) and high specificity (98.81%) compared to ZN staining, which had a sensitivity of 84.85% and specificity of 99.21%. GeneXpert's ability to detect smear-negative TB cases and identify RIF resistance is particularly noteworthy, making it valuable for diagnosing MDR-TB. Early detection and treatment of both drug-sensitive and MDR-TB are crucial for effective TB control and preventing the spread of this highly communicable disease. The findings suggest that GeneXpert is a more reliable and rapid diagnostic tool for TB, supporting its broader implementation to improve diagnostic accuracy and patient outcomes.
